# Lunatic Asylum in Indiana

**Published:** 1844-05

**Authors:** 


### LUNATIC ASYLUM IN INDIANA.

A correspondent informs us that the General Assembly of Indiana, at its last session, authorized a tax of 1 per cent., for the purpose of erecting an Asylum for the Insane. Ere long, therefore, we may hope to see an institution worthy of that young but noble commonwealth.

This action on the part of the Legislature is due mainly to the strenuous exertions of Dr. John Evans, of Fountain county. We have before us now a strong and eloquent address which he delivered by invitation before the Committee on Education of the House of Representatives, and the public, on the 25th of December last. Dr. E., at all sacrifice of time and labor, has not ceased, year after year, to press this matter upon the consideration of the Assembly, and we rejoice that his exertions have at length been crowned with success. We trust that when the proper time arrives, he will consent to take charge of the asylum; in his hands it could not fail to take rank immediately among the first institutions of the country.

